# Supplementary material for: Characterization of Synovial Fluid Components: Albumin-Chondroitin Sulfate Interactions Seen through Molecular Dynamics
Source: Materials (Basel). 2022 Oct 6;15(19):6935. doi: 10.3390/ma15196935 (PMC9572199; doi:10.3390/ma15196935)
Supplement: Supplementary file 1 [file materials-15-06935-s001.zip › materials-1869128-supplementary.pdf]

materials MDPI

Supplementary Materials

Supplementary materials to: Characterization of synovial fluid components: albumin-chondroitin sulfate interactions seen through molecular dynamics

Natalia Kruszewska [1,*], Adam Mazurkiewicz [2], Grzegorz Szala [2], Małgorzata Słomion [3]

[1] Institute of Mathematics and Physics, Bydgoszcz University of Science and Technology, Kaliskiego 7 Street, 85-796 Bydgoszcz, Poland
[2] Faculty of Mechanical Engineering, Bydgoszcz University of Science and Technology, Kaliskiego 7 Street, 85-796 Bydgoszcz, Poland
[3] Faculty of Management, Bydgoszcz University of Science and Technology, Kaliskiego 7 Street, 85-796 Bydgoszcz, Poland
[*] Correspondence: nkruszewska@pbs.edu.pl

Table S1. Lists of the best binding energy obtained in each cluster after molecular docking of CS-4 to HSA.

Docking rank HAS:CS-4	Bind.energy (kcal/mol)	Dissoc. Constant (pM)	Contacting receptor residues
1	3.734	1832068480	PRO 113; ARG 114; LEU 115; VAL 116; ARG 117; PRO 118; ASP 173; ALA 176; LEU 179; PRO 180; LYS 181; ASP 183; GLU 184; ARG 186; ASP 187; PHE 395; GLU 396; GLN 397; LEU 398; GLY 399; GLU 400; TYR 401; LYS 402; LYS 432; SER 435; LYS 436; CYS 438; LYS 439; ALA 511; ASP 512; ILE 513; CYS 514; THR 515; LEU 516; SER 517; GLU 518; LYS 519; GLU 520; ARG 521; GLN 522; LYS 525; ALA 552; ALA 553; VAL 555; GLU 556; LYS 557; CYS 558; CYS 559; LYS 560; ASP 562; ASP 563; LYS 564; GLU 565; PHE 568
2	3.608	2266214144	PHE 156; LYS 159; ARG 160; ALA 163; GLU 167; PRO 180; LYS 181; GLU 184; ASP 187; GLU 188; ALA 191; LYS 195; ARG 218; ARG 222; GLU 277; LYS 281; GLU 285; HIS 288; CYS 289; GLU 292; VAL 293; GLU 294; ASN 295; GLN 390; GLU 393; LEU 394; PHE 395; GLU 396; GLN 397; LEU 398; GLU 400; TYR 401; LYS 402; GLN 404; ARG 410; ARG 428; LYS 432; SER 435; LYS 436; CYS 437; LYS 439; HIS 440; LYS 444; CYS 448; GLU 518; LYS 519; GLN 522; LYS 525; LYS 541; GLU 542; GLN 543; LYS 545; ALA 546; VAL 547; ASP 549; ASP 550; ALA 552; ALA 553; GLU 556; ALA 582
3	3.588	2344018944	GLN 33; PRO 35; PHE 36; GLU 37; THR 83; ASN 111; LEU 112; PRO 113; LEU 115; VAL 116; ARG 117; PRO 118; GLU 119; VAL 122; ALA 126; LYS 137; TYR 140; GLU 141; ARG 144; ARG 145; ASP 173; ALA 176; LEU 179; PRO 180; LYS 181; ASP 183; GLU 184; ARG 186; ASP 187; PHE 395; GLU 396; GLY 399; GLU 400; TYR 401; LYS 402; SER 435; LYS 436; CYS 438; LYS 439; CYS 514; THR 515; LEU 516; SER 517; GLU 518; LYS 519; ARG 521; GLN

			522; LYS 525; LYS 545; ASP 549; ASP 550; ALA 552; ALA 553; VAL 555; GLU 556; LYS 557; CYS 559; LYS 560
4	3.526	2602601728	SER 5 ; ALA 8 ; HIS 9 ; ARG 10; LYS 12; ASP 13; LEU 14; GLY 15; ASN 18; VAL 54; ALA 55; GLU 57; LYS 159; GLU 208; ARG 209; LYS 212; VAL 216; PHE 228; ALA 229; GLU 230; SER 232; LYS 233; THR 236; ASP 237; THR 239; LYS 240; THR 243; ASP 256; ASP 259; LEU 260; LYS 262; TYR 263; CYS 265; GLU 266; ASN 267; GLN 268; ASP 269; SER 270; LYS 276; CYS 279; GLU 280; PRO 282; LEU 283; LEU 284; LYS 286; ASP 324; VAL 325; GLY 328
5	3.517	2642438144	SER 5 ; GLU 6 ; ALA 8 ; HIS 9 ; ARG 10; GLN 32; GLN 33; GLU 57; SER 58; ASN 61; ASP 63; LYS 64; SER 65; HIS 67; THR 68; ASP 72; CYS 75; ARG 81; TYR 84; GLY 85; GLU 86; MET 87; ASP 89; CYS 90; CYS 91; ALA 92; LYS 93; GLN 94; GLU 95; PRO 96; GLU 97; ARG 98; GLU 100; CYS 101; LEU 103; GLN 104; HIS 105; LYS 106; ASP 107; ASP 108; ASN 109; PRO 110; ASN 111; LEU 112; TYR 148; ARG 197; LEU 203; GLN 204; LYS 205; GLY 207; GLU 208; LYS 240; THR 243; GLU 244; HIS 247; ASP 249; GLU 252; ASP 256; GLU 465; LYS 466; THR 467
6	3.496	2737776640	PHE 156; ARG 160; ALA 164; LYS 181; GLU 184; LEU 185; ASP 187; GLU 188; ALA 191; LYS 195; ARG 218; ARG 222; PRO 224; LYS 225; SER 273; LYS 274; LYS 276; GLU 277; LYS 281; HIS 288; GLU 292; VAL 293; GLU 294; ASN 295; ASP 296; GLU 297; MET 298; ALA 300; ASP 301; LEU 302; PRO 303; SER 304; LEU 305; ARG 337; HIS 338; PRO 339; ASP 340; PHE 395; GLU 396; GLY 399; GLU 400; LYS 432; SER 435; LYS 436; CYS 438; LYS 439; HIS 440; PRO 441; GLU 442; ALA 443; LYS 444; ARG 445; PRO 447; CYS 448; LYS 519
7	3.496	2737776640	GLN 33; PRO 35; PHE 36; GLU 37; ASN 109; PRO 110; ASN 111; LEU 112; PRO 113; ARG 114; LEU 115; VAL 116; ARG 117; PRO 118; GLU 119; VAL 120; VAL 122; THR 125; ALA 126; ASP 129; ASN 130; THR 133; PHE 134; LYS 137; TYR 140; GLU 141; ARG 144; ARG 145; ALA 172; ASP 173; ALA 175; ALA 176; LEU 179; PRO 180; ASP 183; LEU 398; GLY 399; GLU 400; TYR 401; LYS 402; PRO 421; SER 435; THR 515; LEU 516; SER 517; GLU 518; LYS 519; ARG 521; GLN 522; LYS 525; LYS 545; ASP 549; ALA 552; VAL 555; GLU 556; CYS 559; LYS 560; ASP 562
8	3.493	2751674368	PHE 156; LYS 159; ARG 160; ALA 163; GLU 167; LYS 181; GLU 184; ASP 187; GLU 188; ALA 191; LYS 195; ARG 218; ARG 222; LYS 281; GLU 285; HIS 288; CYS 289; GLU 292; VAL 293; GLU 294; ASN 295; PHE 395; GLN 397; LEU 398; GLY 399; GLU 400; TYR 401; LYS 402; GLN 404; ARG 428; LYS 432; SER 435; LYS 436; CYS 437; LYS 439; HIS 440; LYS 444; CYS 448; GLU 518; LYS 519; GLN 522; LYS 525; LYS 541; GLU 542; GLN 543; LYS 545;

			ALA 546; VAL 547; MET 548; ASP 549; ASP 550; ALA 552; ALA 553; VAL 555; GLU 556; LYS 560; ALA 578; ALA 582
9	3.407	3181529088	GLN 33; PRO 35; PHE 36; GLU 37; ASP 38; THR 79; LEU 80; ARG 81; GLU 82; THR 83; TYR 84; GLY 85; GLU 86; ALA 88; ASP 89; ALA 92; LYS 93; GLU 97; GLU 100; CYS 101; LEU 103; GLN 104; HIS 105; LYS 106; ASP 107; ASP 108; ASN 109; PRO 110; ASN 111; LEU 112; PRO 113; ARG 114; LEU 115; VAL 116; PRO 118; GLU 119; VAL 122; LYS 137; TYR 140; GLU 141; ARG 144; ARG 145; LEU 203; GLN 204; LYS 205; PHE 206; GLY 207; GLU 208; ARG 209; THR 243; HIS 247; VAL 418; SER 419; THR 420; PRO 421; THR 422; GLU 425; LEU 463; HIS 464; GLU 465; LYS 466; THR 467; THR 474; THR 478; LYS 500; THR 506; PHE 507; THR 508; HIS 510; ASP 512; THR 515; GLU 520; ILE 523; LYS 524; THR 527; GLU 531; LYS 534
10	3.302	3798410496	ARG 114; LEU 115; VAL 116; ARG 117; VAL 120; ALA 175; ALA 176; LEU 179; PRO 180; ASP 183; GLU 184; ARG 186; ASP 187; PHE 395; GLU 396; LEU 398; GLY 399; GLU 400; TYR 401; LYS 402; ARG 428; SER 435; LYS 436; CYS 438; LYS 439; ALA 511; ASP 512; CYS 514; THR 515; LEU 516; SER 517; GLU 518; LYS 519; GLU 520; ARG 521; GLN 522; LYS 525; ASP 549; ALA 552; ALA 553; VAL 555; GLU 556; CYS 558; CYS 559; LYS 560; ALA 561; ASP 562; ASP 563; LYS 564; GLU 565; PHE 568

Table S2. Lists of the best binding energy obtained in each cluster after molecular docking of CS-4 to HSA..

Docking rank HAS:CS-6	Bind.energy (kcal/mol)	Dissoc. constant (pM)	Contacting receptor residues
1	4.356	641223872	GLN 32; GLN 33; ARG 81; GLU 82; THR 83; TYR 84; GLY 85; GLU 86; MET 87; ASP 89; CYS 90; ALA 92; LYS 93; GLU 100; CYS 101; GLN 104; HIS 105; ASP 107; ASN 109; PRO 110; ASN 111; LEU 112; LEU 203; GLN 204; LYS 205; PHE 206; GLY 207; GLU 208; ARG 209; ALA 210; THR 243; HIS 247; PRO 416; GLN 417; SER 419; THR 420; PRO 421; HIS 464; GLU 465; LYS 466; THR 467; PRO 468; VAL 469; SER 470; ASP 471; THR 474; THR 478; GLU 479; ASP 494; THR 496; TYR 497; VAL 498; LYS 500; GLU 501; PHE 502; ASN 503; ALA 504; GLU 505; THR 506; PHE 507; THR 508; PHE 509; HIS 510; GLU 531; LYS 534; PHE 568; ALA 569; GLU 570; GLY 572; LYS 573; VAL 576

2	4.354	643392064	GLN 32; GLN 33; ARG 81; GLU 82; THR 83; TYR 84; GLY 85; GLU 86; GLU 100; GLN 104; HIS 105; ASN 109; PRO 110; ASN 111; LEU 112; PRO 113; ARG 114; LEU 115; ARG 145; LEU 203; GLN 204; LYS 205; PHE 206; GLY 207; GLU 208; ARG 209; ALA 210; THR 243; HIS 247; PRO 416; GLN 417; SER 419; THR 420; PRO 421; THR 422; GLU 425; HIS 464; GLU 465; LYS 466; THR 467; PRO 468; VAL 469; SER 470; ASP 471; ARG 472; THR 474; LYS 475; THR 478; GLU 479; THR 496; TYR 497; VAL 498; LYS 500; GLU 501; PHE 502; ASN 503; ALA 504; GLU 505; THR 506; THR 508; PHE 509; HIS 510; GLU 531; LYS 534; ALA 569; LYS 573; VAL 576
3	4.348	649940736	SER 5; GLU 6; ALA 8; HIS 9; ARG 10; PHE 11; LYS 12; ASP 13; GLY 15; GLU 16; GLU 17; ASN 18; LYS 51; VAL 54; ALA 55; GLU 57; LYS 159; LYS 162; ALA 163; THR 166; GLU 167; LYS 233; ASP 237; LYS 240; ASP 255; ASP 256; ASP 259; LEU 260; LYS 262; TYR 263; GLU 266; ASN 267; GLN 268; LYS 276; CYS 279; GLU 280; LYS 281; PRO 282; LEU 283; LEU 284; GLU 285; LYS 286
4	4.347	651038592	GLN 32; GLN 33; ARG 81; GLU 82; THR 83; TYR 84; GLY 85; GLU 86; MET 87; GLU 100; GLN 104; HIS 105; ASP 107; PRO 110; ASN 111; LEU 203; GLN 204; LYS 205; PHE 206; GLY 207; GLU 208; ARG 209; ALA 210; THR 243; HIS 247; PRO 416; GLN 417; SER 419; THR 420; PRO 421; HIS 464; GLU 465; LYS 466; THR 467; PRO 468; VAL 469; ASP 471; THR 474; THR 478; GLU 479; THR 496; TYR 497; VAL 498; LYS 500; GLU 501; PHE 502; ASN 503; ALA 504; GLU 505; THR 506; PHE 507; THR 508; PHE 509; HIS 510; GLU 531; LYS 534; ALA 569; GLU 570; GLY 572; LYS 573; VAL 576
5	4.339	659888896	GLN 32; GLN 33; ARG 81; GLU 82; THR 83; TYR 84; GLY 85; GLU 86; MET 87; GLU 100; GLN 104; HIS 105; ASP 107; ASN 111; ARG 114; LEU 203; GLN 204; LYS 205; PHE 206; GLY 207; GLU 208; ARG 209; ALA 210; THR 243; HIS 247; PRO 416; GLN 417; SER 419; THR 420; PRO 421; HIS 464; GLU 465; LYS 466; THR 467; PRO 468; VAL 469; SER 470; ASP 471; THR 474; THR 478; GLU 479; THR 496; TYR 497; VAL 498; LYS 500; GLU 501; PHE 502; ASN 503; ALA 504; GLU 505; THR 506; THR 508; PHE 509; HIS 510; ASP 512; LEU 516; GLU 520; GLU 531; LYS 534; ALA 569; GLU 570; GLY 572; LYS 573; VAL 576

6	4.322	679097280	VAL 116; GLU 119; PHE 156; ARG 160; ALA 175; ALA 176; CYS 177; LEU 179; PRO 180; LYS 181; ASP 183; GLU 184; ASP 187; GLU 188; ALA 191; LYS 195; HIS 288; GLU 292; PHE 395; GLU 396; GLN 397; LEU 398; GLY 399; GLU 400; TYR 401; LYS 402; ASN 405; LYS 432; SER 435; LYS 436; CYS 438; LYS 439; ALA 511; ASP 512; CYS 514; THR 515; LEU 516; SER 517; GLU 518; GLU 520; ARG 521; GLN 522; LYS 525; LYS 545; MET 548; ASP 549; ALA 552; VAL 555; GLU 556; CYS 558; CYS 559; LYS 560; ALA 561; ASP 562; ASP 563; LYS 564; GLU 565; PHE 568
7	4.314	688329024	SER 5; GLU 6; HIS 9; ARG 10; SER 58; ALA 59; GLU 60; ASN 61; ASP 63; LYS 64; SER 65; LEU 66; THR 68; ASP 72; GLU 86; ASP 89; ALA 92; LYS 93; GLN 94; GLU 95; PRO 96; GLU 97; GLU 100; CYS 101; LEU 103; GLN 104; HIS 105; LYS 106; ASP 107; LEU 203; GLN 204; LYS 205; PHE 206; GLY 207; GLU 208; ARG 209; ALA 210; LYS 212; THR 236; LYS 240; GLU 244; HIS 247; GLU 252; TYR 319; ALA 320; GLU 321; ALA 322; LYS 323; ASP 324; VAL 325; GLU 358; GLU 465; LYS 466; PRO 468; THR 474; THR 478
8	4.308	695335040	PRO 35; PHE 36; GLU 37; ASP 38; VAL 40; LYS 41; LEU 42; ASN 44; GLU 45; GLU 48; PHE 49; SER 58; ALA 59; GLU 60; ASN 61; ASP 63; LYS 64; SER 65; THR 68; LEU 69; ASP 72; LYS 73; CYS 75; THR 76; VAL 77; THR 79; LEU 80; GLU 82; THR 83; ALA 88; CYS 91; ALA 92; LYS 93; GLN 94; GLU 95; ARG 98; LEU 112; PRO 113; ARG 114; LEU 115; VAL 116; PRO 118; VAL 122; MET 123; ALA 126; ASP 129; ASN 130; GLU 132; THR 133; PHE 134; LYS 136; LYS 137; TYR 140; ARG 144
9	4.274	736404544	PRO 113; ARG 114; VAL 116; ARG 117; PRO 118; GLU 119; VAL 120; ASP 121; VAL 122; LYS 137; ALA 175; ALA 176; LEU 179; PRO 180; ASP 183; GLU 184; ARG 186; ASP 187; GLU 188; GLU 393; PHE 395; GLU 396; GLN 397; LEU 398; GLY 399; GLU 400; TYR 401; LYS 402; LYS 432; GLY 434; SER 435; CYS 438; LYS 439; ARG 445; HIS 510; ALA 511; ASP 512; CYS 514; THR 515; LEU 516; SER 517; GLU 518; LYS 519; GLU 520; GLN 522; LYS 525; GLU 542; GLN 543; LYS 545; ALA 546; ASP 549; CYS 558; CYS 559; LYS 560; ALA 561; ASP 562; ASP 563; LYS 564; GLU 565; PHE 568
10	4.249	768142336	HIS 9; ARG 10; GLU 208; ARG 209; LYS 212; VAL 216; PHE 228; ALA 229; SER 232; LYS 233; VAL 235; THR 236; ASP 237; THR 239; LYS 240; THR 243; ASP 256; ASP 259; MET 298; ASP 301; LEU 302; PRO 303; SER 304; ALA 306; ALA 307; ASP 308; VAL 310; GLU 311; SER 312; LYS 313; ASP 314; CYS 316; LYS 317; ASN 318; TYR 319; ALA 320; GLU 321; ALA 322; LYS 323; ASP 324; VAL 325; PHE 326; MET 329; TYR 334; ARG 337; HIS 338; PRO 339; ASP 340; TYR 341; GLU 354; GLU 358; ASP 365; HIS

367; GLU 368; TYR 370; ALA 371; LYS 372; PHE 374; ASP 375; PHE 377; LYS 378; VAL 381; GLU 382

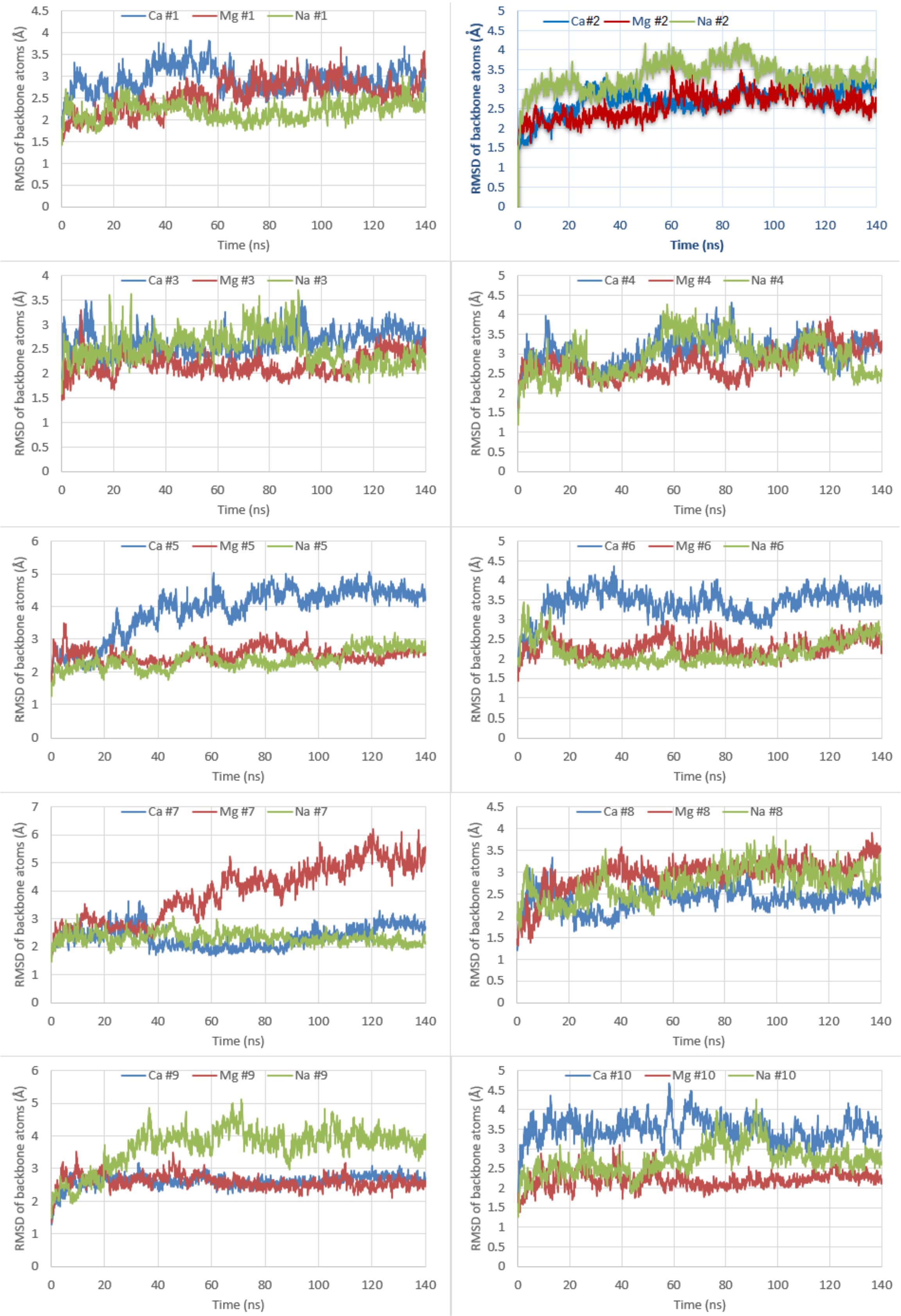

Figure S1. RMSD of backbone atoms as a function of time for HSA:CS-6 complexes.

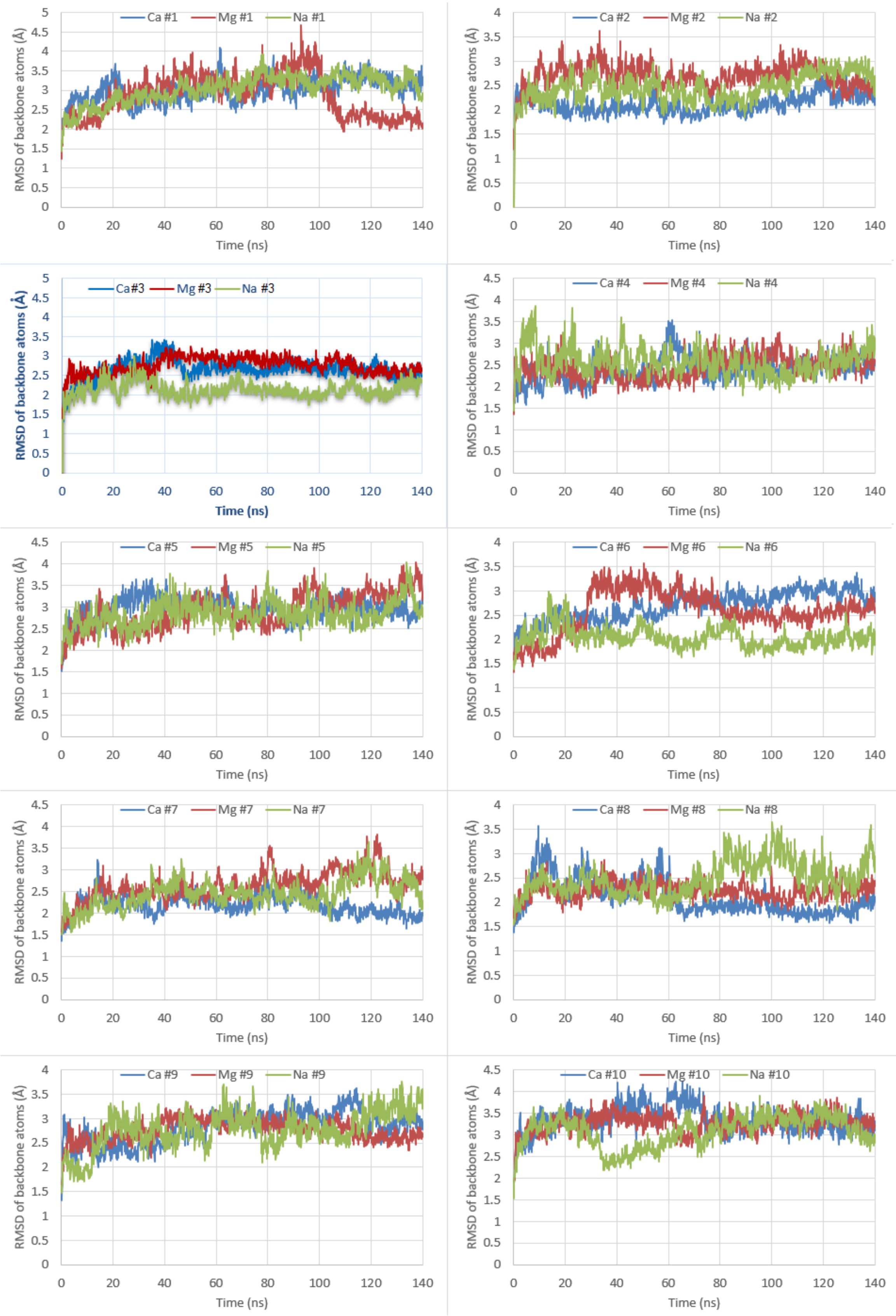

Figure S2. RMSD of backbone atoms as a function of time for HSA:CS-4 complexes.

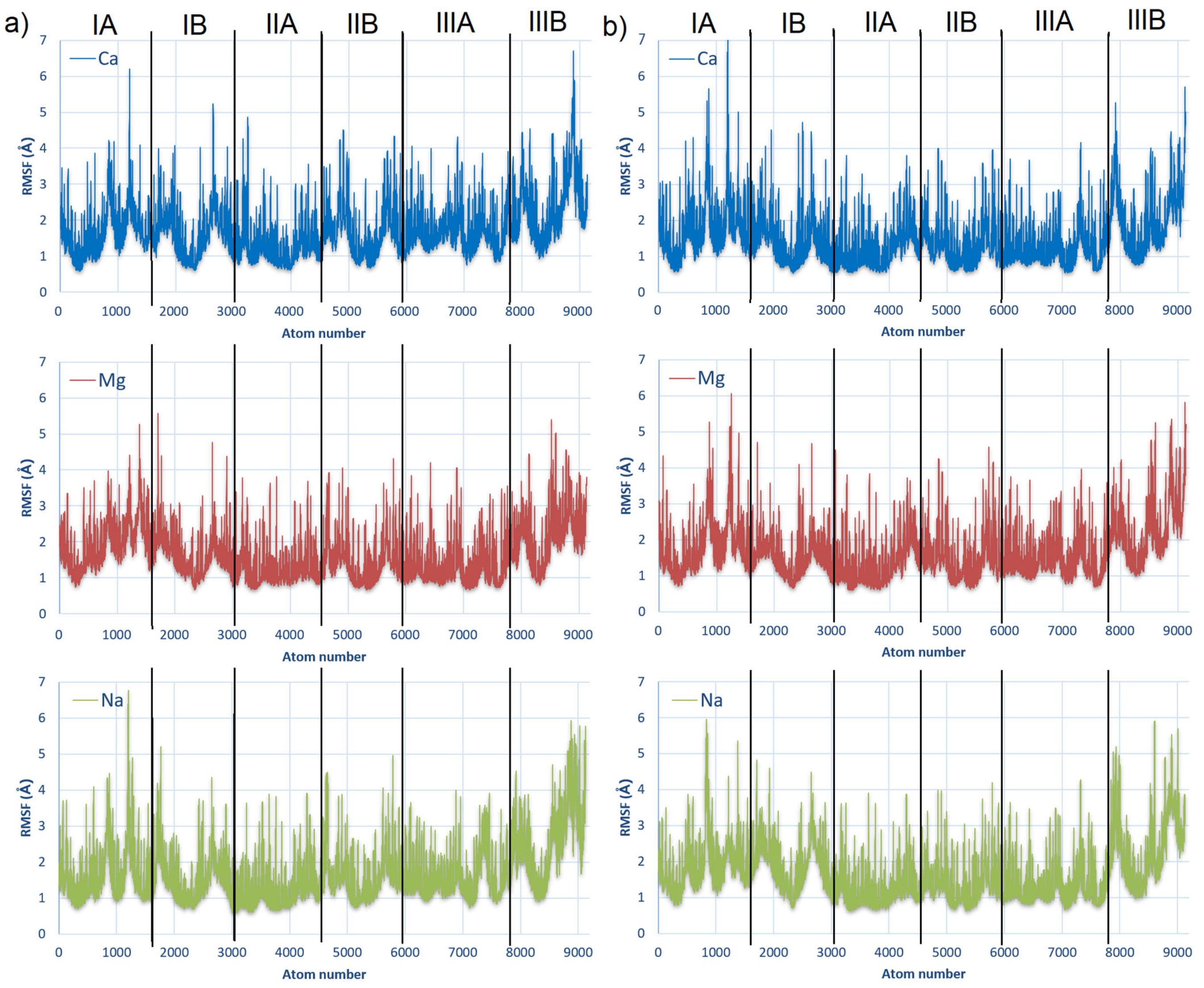

Figure S3. RMSF values for each atom of HSA for best-bound a) #2 HSA:CS-6, b) #3 HSA:CS-4 complexes.

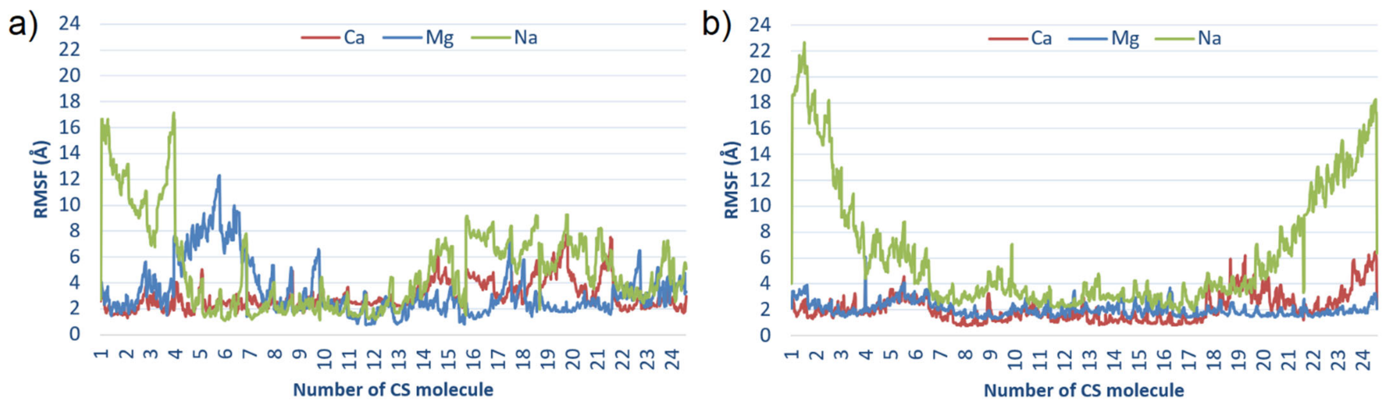

Figure S4. RMSF values for each atom of CS (1180 atoms) for best-bound a) #2 HSA:CS-6, b) #3 HSA:CS-4 complexes.

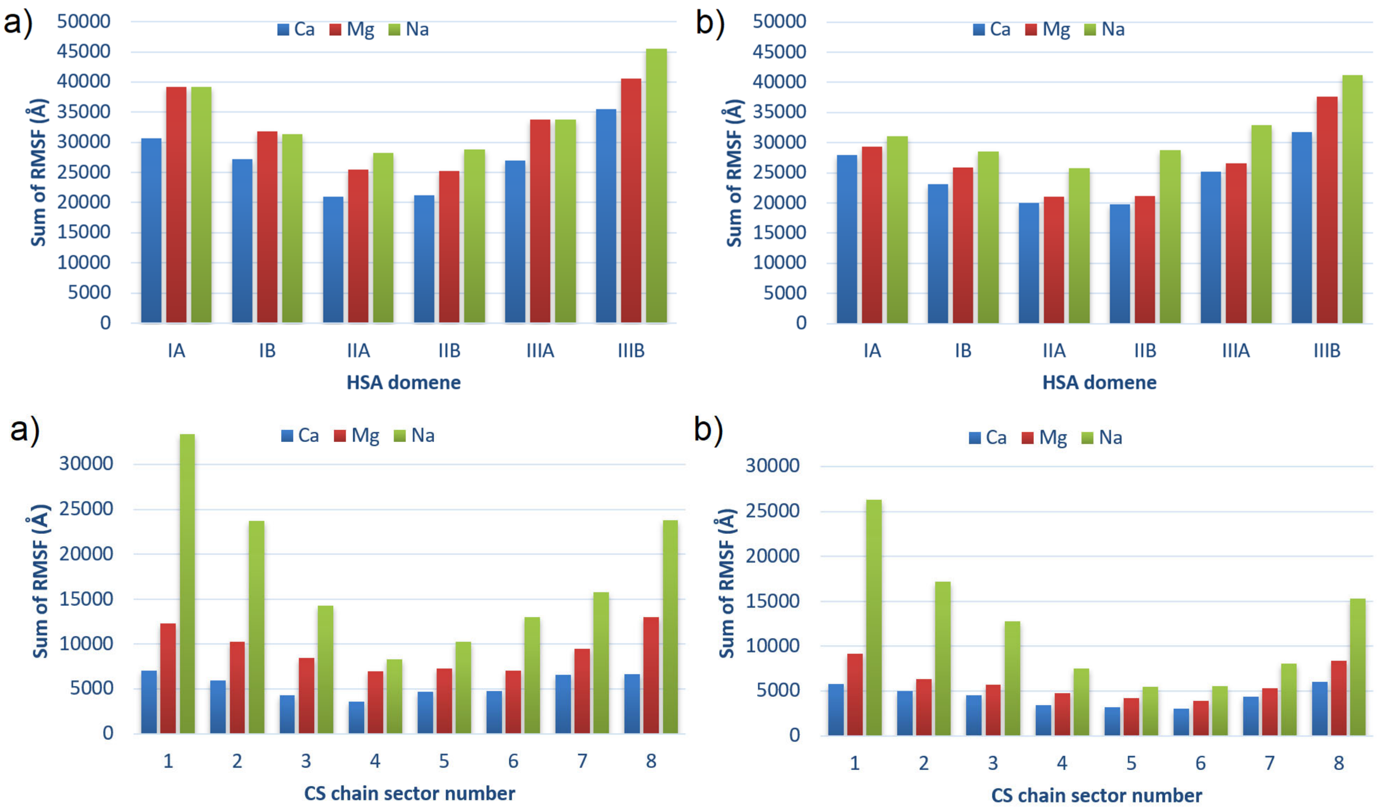

Figure S5. Sum of RMSF for HSA (top) and CS (bottom) for complexes: a) HSA:CS-6, and b) for HSA:CS-4.

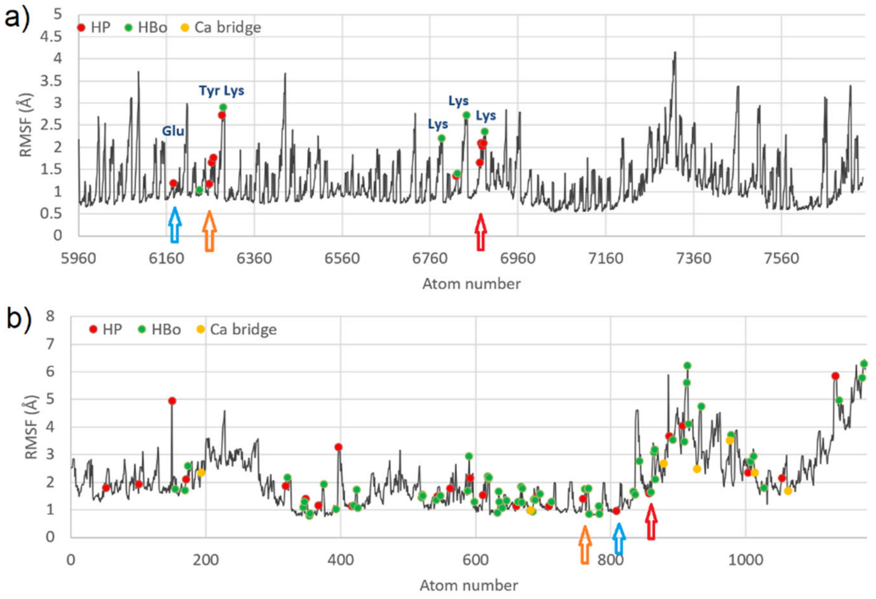

Figure S6. RMSF for IIIA domain of HSA (a) and CS-4 (b) for complex #3 HSA:CS-4. Places of specific interactions are marked with dots.

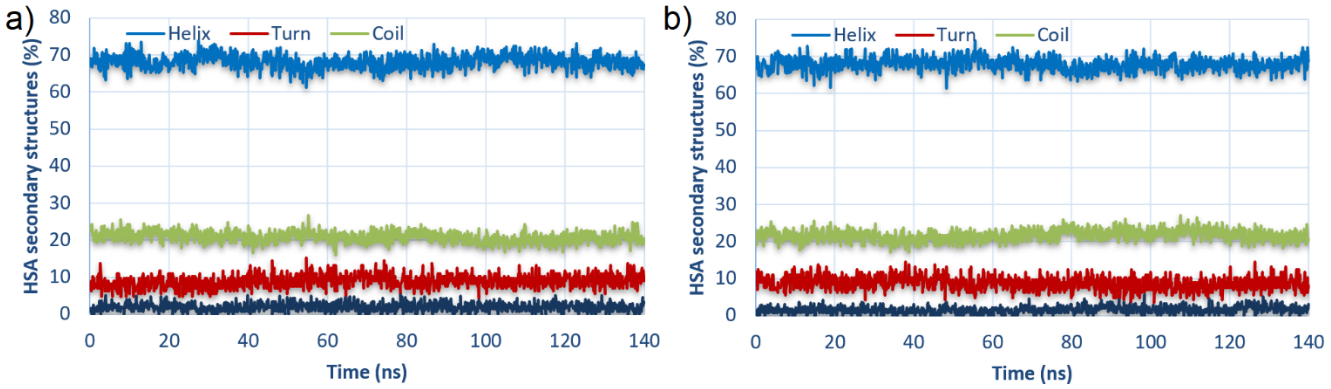

Figure S7. Secondary structure of HSA as a function of time for: a) #2 HSA:CS-6, b) #3 HSA:CS-4 complexes in $CaCl_2$ solution.

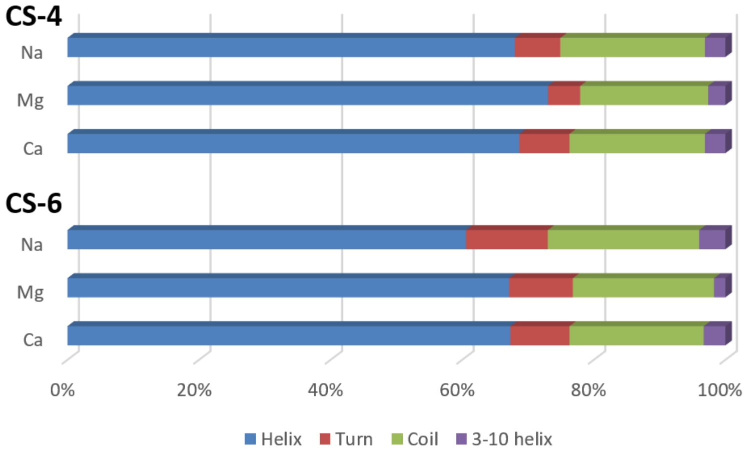

Figure S8. Secondary structure of HSA after 140 ns of MD simulations for best-bound structures.

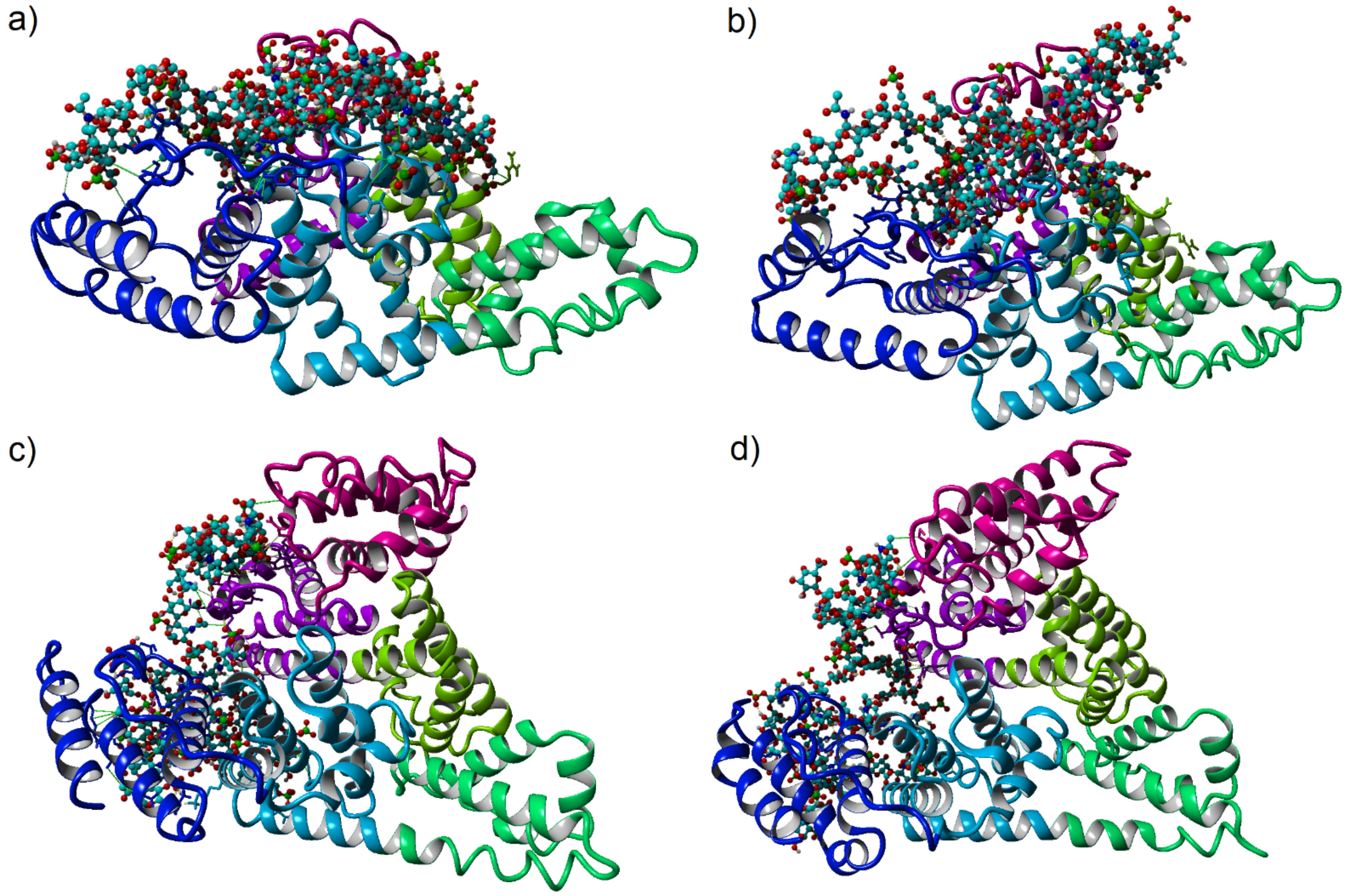

Figure S9. 3D structures of HSA:CS-6 (a and b) and HSA:CS-4 (c and d) for the strongest bound complexes after MD in $MgCl_2$ solution (solution is transparent on the picture). HSA domains are colored as follows: IA-pink, IB-violet, IIA-light green, IIB-green, IIIA-light blue, IIIB-blue. In CS-4 and CS-6, light blue atoms represent carbon, dark blue nitrogen, red oxygen, green sulfur and white hydrogen. Snapshots done using YASARA software (a and c) before and (b and d) after 140 ns of MD simulations.

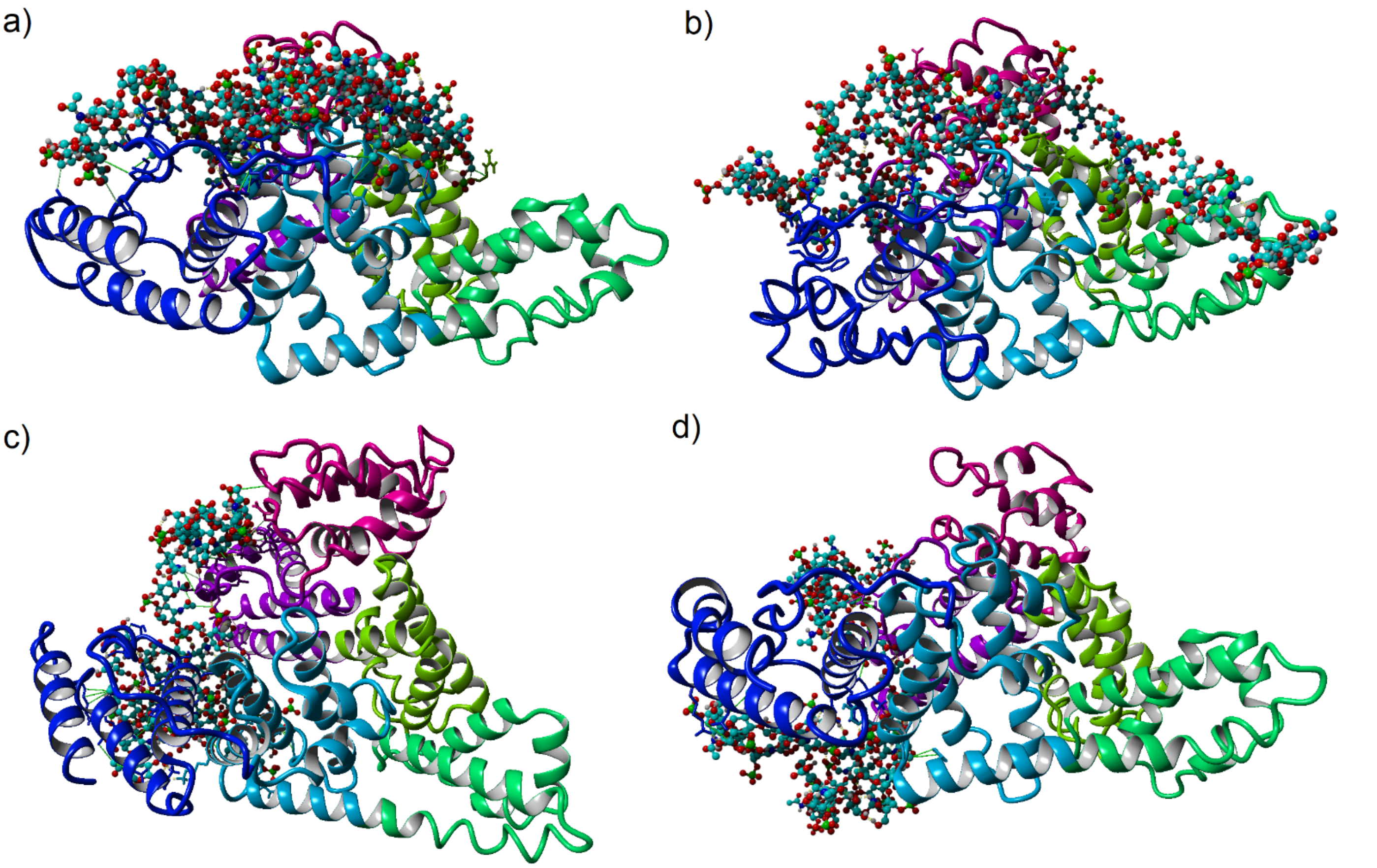

Figure S10. 3D structures of HSA:CS-6 (a and b) and HSA:CS-4 (c and d) for the strongest bound complexes after MD in NaCl solution (solution is transparent on the picture). HSA domains are colored as follows: IA-pink, IB-violet, IIA-light green, IIB-green, IIIA-light blue, IIIB-blue. In CS-4 and CS-6, light blue atoms represent carbon, dark blue nitrogen, red oxygen, green sulfur and white hydrogen. Snapshots done using YASARA software (a and c) before and (b and d) after 140 ns of MD simulations.

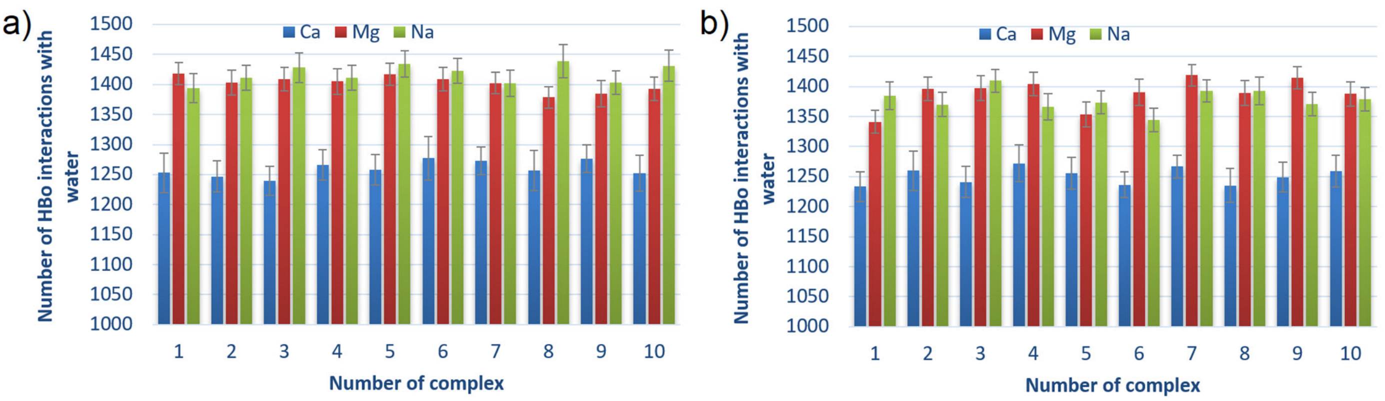

Figure S11. Number of HBo between a) HSA:CS-6 or b)HSA:CS-4, and water molecules. Complexes are sorted from lowest to highest energy of binding after MD simulationsl; thus, first is strongest bound (cf. Table 1). Error bars present doubled STD.
